# Locally controlled release of immunosuppressive promotes survival of transplanted adult spinal cord tissue

**DOI:** 10.1093/rb/rbac097

**Published:** 2022-12-05

**Authors:** Ziqiang Wang, Ya Li, Chenxuan Sun, Pukong Cui, Yuanyuan Han, Tong Wu, Bai Xu, Can Zhang, Liyang Shi, Jianwu Dai

**Affiliations:** College of Biology, Hunan University, Changsha 410000, China; College of Biology, Hunan University, Changsha 410000, China; College of Biology, Hunan University, Changsha 410000, China; College of Biology, Hunan University, Changsha 410000, China; College of Biology, Hunan University, Changsha 410000, China; College of Biology, Hunan University, Changsha 410000, China; State Key Laboratory of Molecular, Developmental Biology, Institute of Genetics and Developmental Biology, Chinese Academy of Sciences, Beijing 100101, China; College of Biology, Hunan University, Changsha 410000, China; College of Biology, Hunan University, Changsha 410000, China; College of Biology, Hunan University, Changsha 410000, China; State Key Laboratory of Molecular, Developmental Biology, Institute of Genetics and Developmental Biology, Chinese Academy of Sciences, Beijing 100101, China

**Keywords:** spinal cord injury, adult spinal cord tissue transplantation, immune rejection, controlled release, tacrolimus

## Abstract

Transplantation of adult spinal cord tissue (aSCT) is a promising treatment for spinal cord injury (SCI) basing on various types of neural cells and matrix components inside aSCT. However, long-term systemic administration of immunosuppressors (e.g. tacrolimus, TAC) is required for the survival of allogeneic tissue, which often associated with severe side effects such as infection, liver damageand renal failure. In this study, a triglycerol monostearate (TGM)-based TAC delivery system (e.g. TAC@TGM) with high drug loading concentration was developed, which possessed injectable properties as well as sustainable and immune-responsive drug release behaviors. In complete transected SCI model, locally injected TAC@TGM could reduce the infiltration of inflammation cells, enhance the survival of transplanted aSCT (e.g. Tuj-1^+^ and NF^+^ neurons) and promote the recovery of locomotor function. Moreover, controlled release of TAC by TAC@TGM attenuated side effects of TAC on liver and kidneys compared with traditional systemic administration. More importantly, the developed TAC@TGM system provided a facile single dose of long-term immunosuppressive effect not just for aSCT transplantation, but also for other tissue/organ and cell transplantations.

## Graphical abstract

**Figure:**
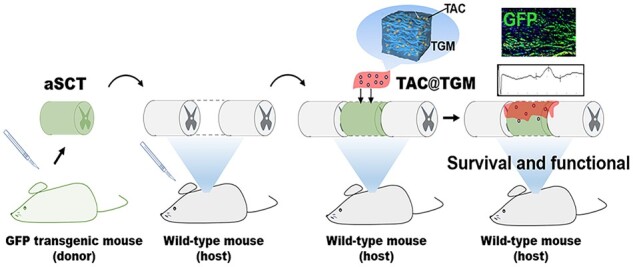

## Introduction

Spinal cord injury (SCI) significantly damages the central nervous system and causes sensory and motor ability lose below the SCI area [1]. It is estimated that more than 27 million people suffer from SCI worldwide, with an annual increase of 930 000 new cases [2]. Unfortunately, it is still a big challenge to fully restore the structure and function of injured spinal cord [3]. Transplantation of spinal cord tissue (SCT) is promising for the repair of SCI, which had been first reported in 1983 [4]. Axonal connectivity was observed between the host and donor tissues, some host fibers extended into the transplantation area and generated functional connections between the donor and host tissues [5–7]. Another excellent study by Iwashita [8] also reported that the transplanted SCT exhibited robust axonal growth and regeneration, which enabled rats walk, run and climb with almost normal hind forelimb coordination. In our previous study, we proved that transplanted adult SCT (aSCT) into SCI sites in rat and canines could promote the recovery of locomotor function [9, 10]. At present, the main obstacle of organ/tissue transplantation is immune rejection [11]. In clinical, immunosuppressive drugs such as tacrolimus (TAC) were widely applied through systemic administration to promote the survival of transplanted organs and tissues [12]. However, perennial systemic administration of TAC often led to severe complications, such as infection, liver damage, renal failure and malignancy tumor [13–19].

Local drug delivery approaches can reduce drug blood concentration through controllable release tech [20], as well as increase *in situ* drug concentration to obtain single dose for long-term therapeutic effect [21]. It was reported that TAC encapsulated in poly (lactic-co-glycolic) acid (PLGA) microspheres and suspended in fibrin hydrogel realized locally drug controlled release, enhanced axon regeneration after nerve autografts and allografts transplantation and effectively prevented immune rejection of nerve allografts [22]. Moreover, TAC loading PLGA nanoparticle encapsulating self-assembling peptide (RADA16) hydrogel resulted in localized TAC release, thereby improving the survival of transplanted cells and finally generated efficiently tissue regeneration [23]. Sustainable TAC-releasing peptide hydrogels disassembled by protein tyrosine kinase that is released by T cells, which significantly extended the rat of median survival time into 22 days in liver transplantation model comparing with that of 13 days after receiving conventional TAC treatment [24]. However, due to the hydrophobicity of TAC, the low drug loading efficiency and uncontrolled release behaviors are still major challenges to prepare long-term TGM release system with single-dose administration.

**Figure 1.**
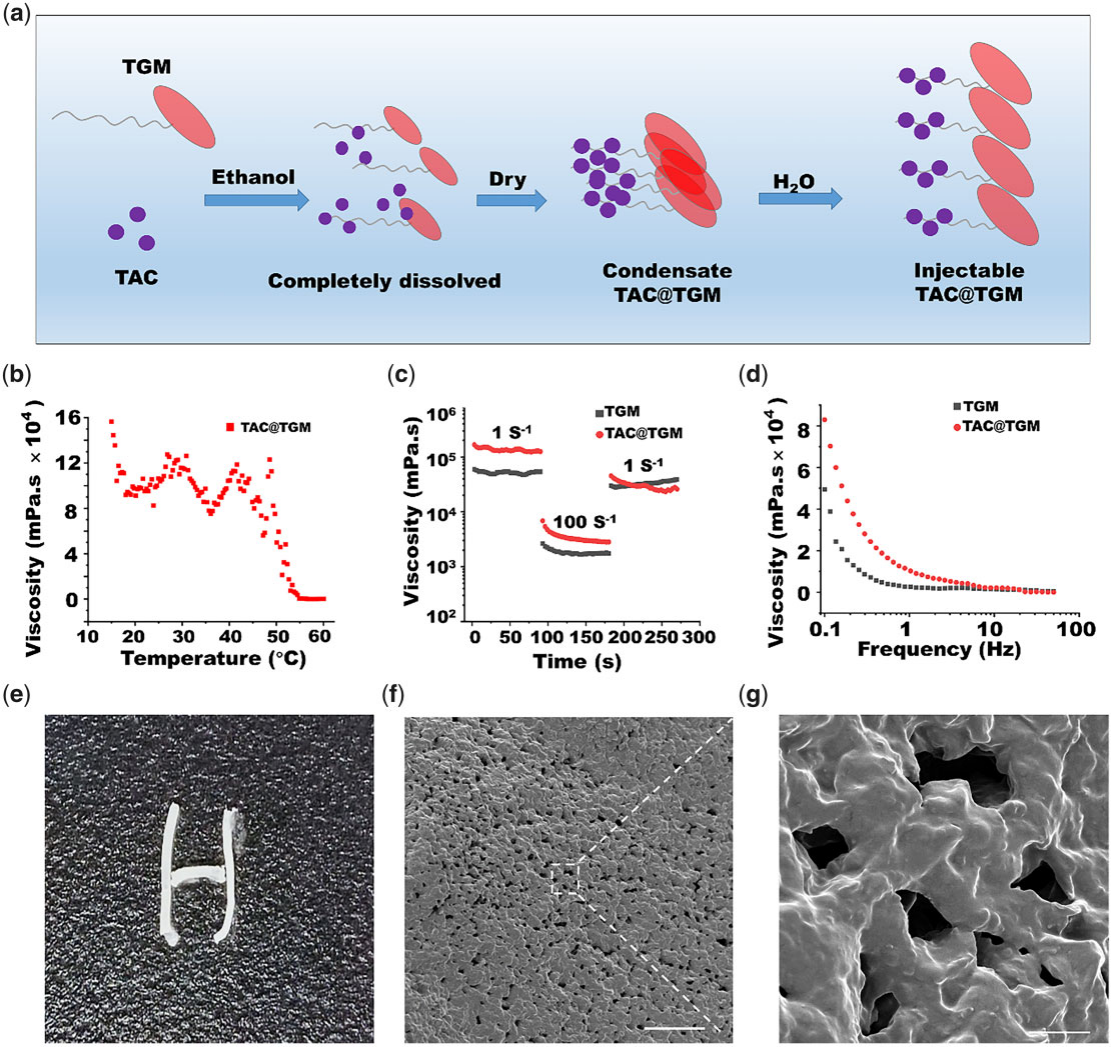
The preparation and characterization of TAC@TGM. (**a**) Schematic diagram of preparing TAC@TGM. First step: TAC and TGM were completely dissolved in absolute ethanol, and a condensate of TAC@TGM was obtained after drying; second step: the condensate of TAC@TGM was blended with water to obtain the injectable TAC@TGM. (**b**–**d**) The viscosity of TAC@TGM was measured at the indicated temperatures, times and frequencies. (**e**) Injection of TAC@TGM from a syringe. (**f**, **g**) Scanning electron microscopy images of TAC@TGM. Scale bar, 100 μm (f) and 10 μm (g).

Triglycerol monostearate (TGM) is an amphiphile with both hydrophilic and hydrophobic groups. The hydrophilic site includes multiple hydroxyl groups, permitting TGM soluble in water, and the hydrogen bond network in hydrophilic groups making TGM self-assemble into gel [25]. The hydrophobic site includes a polymethylene hydrocarbon chain, which can efficiently encapsulate hydrophobic molecules via van der Waals forces [26]. For example, the hydrophobic drug curcumin was encapsulated in TGM for the treatment of traumatic brain injury [27]. In addition, TGM has an ester bond that can be cleaved by proteolytic enzymes, such as matrix metalloproteinases (MMPs), which are produced by macrophages and accumulated significantly under inflammatory conditions [28]. This biodegradable property of the inflammatory response provides a new pathway for drug release. Based on these advantages of TGM, we developed a two-step method to prepare a controlled release system of TAC (TAC@TGM) (Fig. 1a). Briefly, TAC and TGM were completely dissolved in absolute ethanol, and then the ethanol was removed by evaporation to obtain a condensed TAC@TGM. Before using, the condensate of TAC@TGM was blended with water to produce the injectable TAC@TGM. Rheological behavior, TAC release and cytocompatible tests were carried out *in vitro* to evaluate the potential of TAC@TGM as local delivery system. In the *in vivo* experiment, T8–T9 aSCTs from green fluorescent protein (GFP) transgenic mice were transplanted into wild-type C57 BL/6 mice, and TAC@TGM was applied to the surface of transplanted aSCT. Locomotor function restoration, survival of aSCT, immune response and toxic effects on other organs were investigated. In summary, this study developed an inflammatory responsive controlled release system of TAC to reduce local immune rejection and promote the survival and function of aSCT.

## Materials and methods

### TAC@TGM preparation

Briefly, 2 ml of ethanol was added to a glass scintillation vial with 10 mg TAC (Selleck, S5003) and 150 mg TGM (SantaCruz, sc-460912) (6.7%, w/w), and sealed with a screw cap. The vial was heated to 60°C until TGM and TAC were dissolved, after that, removed the screw cap and the vial was placed in a 50°C oven, dried for 48 h until weight no longer changed. Then blended with 200 μl of deionized water. After 30 min, the mixture formed a stable structure. A scanning electron microscope (SEM) (TESCAN, Czech Republic) was used to observe the morphology of TAC@TGM after freeze drying. Additionally, the average pore size was calculated from the average of 10 pore sizes in 3 randomly selected areas. The concentration of the drug was calculated by the following formula: Concentration (mg/ml) = TAC mass/total volume of material.

### The viscosity properties

The viscosity properties of TGM or TAC@TGM were measured on a rheometer (Anton Paar, MCR-92) using a parallel plate (Φ = 15 mm) under the rotating mode. And the gap of the parallel plate was set to 0.75 mm. The viscosity of TGM and TAC@TGM was measured under the following conditions: frequency range from 0.1 to 50 Hz at 25°C; shear rates from 1 to 100 Hz, repeatedly at a fixed temperature of 25°C and dynamic temperature sweep (15–60°C) at a fixed shear rate of 1 Hz.

### TAC release profile

TAC@TGM (30 mg) samples were placed in 3 ml release media of saline at 37°C. To further verify the release of TAC@TGM under inflammatory conditions, TAC@TGM (30 mg) samples were placed in 3 ml release media contained 2.95 ml saline and 50 μl activated macrophage culture supernatant at 37°C. At predetermined time points, the release media was replaced with 3 ml of new release media. The content of TAC in the release media was measured using a UV spectrophotometer at 208 nm. Preparation of activated macrophage culture supernatant: RAW264.7 cells (Procell, CL-0190) were seeded in 6-well plates at a density of 3 × 10^5^ cell/well and incubated for 12 h. Then changed medium with 200 ng/ml Lipopolysaccharide (LPS) (Sigma-Aldrich, 0111: B4) for 24 h.

### Neural stem cell culture

The neural stem cell culture was referred to the previous literature [29]. Briefly, 10 newborn mice were sacrificed. The skull was removed. Then the brain and hippocampus were removed and placed in phosphate-buffered saline (PBS). The meninges and blood vessels were removed, the tissue was cut with scissor, then 0.125% trypsin was added (Gibco, 25200056) and the tissue was placed in an incubator at 37°C for 20 min. After 20 min, the digestion was terminated by adding the same amount of serum, and the cells were suspended using neural stem cell proliferation medium. The neural stem cells were incubated in a T75 culture bottle (NEST Biotechnology) in a 5% CO_2_ incubator at 37°C.

### Cytocompatibility assay

Neural stem cells were cultured in medium containing TGM or TAC@TGM extracts for 48 h. Calcein/PI Cell Cytotoxicity Detection Kit (Beyotime, C2015M) was used to conduct live/dead staining. In the cell viability test, neural stem cells in the logarithmic growth phase were seeded on 96-well plates, incubated with medium containing TGM or TAC@TGM extracts for 48 h and measured absorbance values at 450 nm after incubating with CCK-8 reagent (Bimake, B34304) for 2 h. The absorbance value at 450 nm was detected with a microplate reader and the cell viability was calculated with the following formula. Cell survival rate (%) = (OD value of experimental group − OD value of blank group)/(OD value of control group − OD value of blank group) × 100%.

### Surgery procedures

Animal care, housing and experimental protocols were performed in accordance with the standard guidelines approved by the Animal Ethics Committee of Hunan University (Number HNUBIO202203001). Mice (*n* = 20) used in the experiment were females weighing 18–20 g, randomly divided into four groups. The surgery procedure was slightly modified according to the previous report [9]. The GFP-transgenic mice (*n* = 20) (Cyagen Biotechnology Co., Ltd) were used as donors and the wild-type C57BL/6 mice (Hunan SJA Laboratory Animal Co., Ltd) were used as recipients. Briefly, under general anesthesia, a gap wound was created and T7–10 laminae were removed. After that, the spinal cord was exposed, followed by a 2-mm long transection in T8–9. Bleeding was ceased using a gelatin sponge filled up in the transection site. Then, the donor aSCTs of the GFP mice were transplanted into the lesion sites of the wild-type C57BL/6 mice in the correct rostral and caudal orientation. Following treatments, the skin and musculature were closed with sutures. Postoperative antibiotics were given for 3 days and bladder massage care was given twice a day until sacrifice.

### Electrophysiological analysis

MEPs were measured with an electromyography system (Medcom Technology, M-8000C). Two stimulating electrodes were inserted onto scalp and palate, respectively. The recording electrodes were placed on the gastrocnemius muscle of the hind limb, MEPs were recorded after stimulation at 20 mA.

### Behavioral assessment

Basso Mouse Scale (BMS) score was used to access the locomotion recovery after SCI [30]. The hind limbs locomotor function recovery profiles of animals were assessed weekly by three independent observers blinded to the treatments.

### Cell counts in peripheral blood

Mouse blood samples were obtained from retro-ocular veins at the fourth week post-surgery. Samples were collected using heparin anticoagulant tubes and examined using a mouse hematology analyzer (SHINOVA, Hemo 3600V).

### Histology and immunofluorescence staining

The spinal cord samples, heart, liver, spleen, lung and kidney were embedded in Tissue-Tek^®^ O.C.T. (Sakura Finetek Inc.) and then frozen sectioned (10 μm) using a Leica CM1950 cryostat (Leica Camera Inc.). Hematoxylin–eosin (HE) staining was applied for general morphology observation according to the previous method [18]. Image-J software (Wayne Rasband, National Institutes of Health) was used to calculate the percentage of area occupied by inflammatory cells in the visual field. For immunofluorescence: the spinal cord sections were blocked with PBS containing 5% serum, incubated in 0.1% Triton X-100 for 10 min, then incubated with primary antibodies at 4°C overnight. Then incubated with secondary antibodies. The primary and secondary antibodies used in this study were: Beta-tubulin III (Tuj-1) (Abcam, b7751); neurofilament (NF) (Abcam, b207176); inducible nitric oxide synthase (iNOS) (Abcam, ab178945); CD4 (BD Pharmingen, 552775); tumor necrosis factor alpha (TNF-α) (Proteintech, 26405); interleukin-2 (IL-2) (Proteintech, 26156) and goat anti-rabbit IgG, Alexa Fluor 546 (Invitrogen, A-11035) and goat anti-rat IgG Alexa Fluor 546 (Invitrogen, A-11081). 4’,6-diamidino-2-phenylindole (DAPI) (Sigma, f6057) was used for nuclear staining. All images were captured using a confocal microscope (Nikon, TI-E+A1RMP+N-STORM). The immunofluorescence staining analysis was based on the number of positive cells and the total number of cells in the selected area, DAPI-stained nuclei were used for the count.

### Determination of serum biochemical indicators

Blood was collected from mice using anticoagulant centrifuge tubes. Then the blood was centrifuged at 1000 × g for 10 min to obtain serum. The serum was stored at −80°C. Alanine aminotransferase (ALT) Assay Kit (Nanjing Jiancheng Bio., C009-2-1) was used to determine the level of ALT in serum. Aspartate transaminase (AST) Assay Kit (Nanjing Jiancheng Bio., C010-2-1) was used to determine the level of AST in serum. Creatinine Assay kit (Nanjing Jiancheng Bio., C011-2-1) was used to determine the level of creatinine in serum. Urea Assay Kit (Nanjing Jiancheng Bio., C013-2-1) was used to determine the level of blood urea nitrogen (BUN) in serum.

### Statistical analysis

The data were analyzed by SPSS statistical package 18.0 software (IBM, USA). Data were presented as mean values ± standard deviation. The one-way analysis of variance followed by Student–Newman–Keuls (S–N–K) test was performed to assess multiple independent groups. The independent sample *t*-test was used to compare two independent samples. *P < 0.05; **P < 0.01; ***P < 0.001; ns indicates no statistically significant.

## Results and discussion

### Fabrication and characterization of TAC@TGM

A two-step method (Fig. 1a) was developed to prepare a TAC-controlled release system based on the lipophilic character of TGM and TAC. Briefly, TAC and TGM were completely dissolved in absolute ethanol at 60°C, so that TAC was inserted into the hydrophobic group in TGM. The resulting condensate of TAC@TGM with uniform distribution of TAC was obtained after the ethanol was completely removed by drying. The condensate of TAC@TGM was then mixed with water (with 1:1 of mass:volume ratio) to obtain the injectable TAC@TGM by self-assembly of TGM.

The rheology characters including viscosity–temperature curve, viscosity–time curve and viscosity–frequency curve of TAC@TGM were determined. Viscosity–temperature curve showed that TAC@TGM could maintain a stable morphology below 50°C, which enabled its application at body temperature. The viscosity of TAC@TGM dropped sharply between 50°C and 60°C (Fig. 1b) and led to a phase transition from solid to liquid. The thixotropic property of TAC@TGM was demonstrated by the recovery of viscosity (Fig. 1c). The viscosity significantly decreased from low shear rate (1 S^−1^) into high one (100 S^−1^), while the samples viscosity recovered upon the shear rate decreasing into low value (1 S^−1^). These results proved that TAC@TGM and TGM have good thixotropic characters, the drug additions did not affect the internal structure of TGM. In the continuous viscosity–shear rate curve, the viscosity of both TAC@TGM and TGM gradually decreased with the increase of the shear rate (Fig. 1d), indicating their shear thinning property and injectability. Based on the viscosity properties, TAC@TGM could be injected through a narrow needle, which providing a non-invasive drug delivery strategy for TAC (Fig. 1e). SEM observation showed that TAC@TGM was porous and uniform, with an average pore diameter of 11.68 ± 3.83 μm (Fig. 1f and g). This ensured TGM as an environmental responsive release system with both the entry of inflammatory factors (e.g. MMPs) and the release of TAC. To determine whether TAC@TGM could be easily delivered to target site, we measured the injection force at various injection conditions (a 1 cc syringe or 2.5 cc syringe with different sizes of needle, 18G, 23G and 25G) by referring to the method of Lee *et al.* [31]. The TAC@TGM could be injected stably and exhibited varying levels of injection force with different syringe sizes. The force required to inject TAC@TGM increased with the increase of syringe volume (2.5 cc > 1 cc syringes) as well as needle sizes (25G > 23G > 18G) (Supplementary Fig. S1a and b). In addition, we measured the injection forces of 25 mg/ml TAC@TGM (TAC@TGM25), 50 mg/ml TAC@TGM (TAC@TGM50) and 75 mg/ml TAC@TGM (TAC@TGM75), it was observed that the force decreased with the increase of the TAC concentration. The results showed that the increasing TAC concentration would decrease the injection force of TAC@TGM without affecting its injectability.

**Figure 2.**
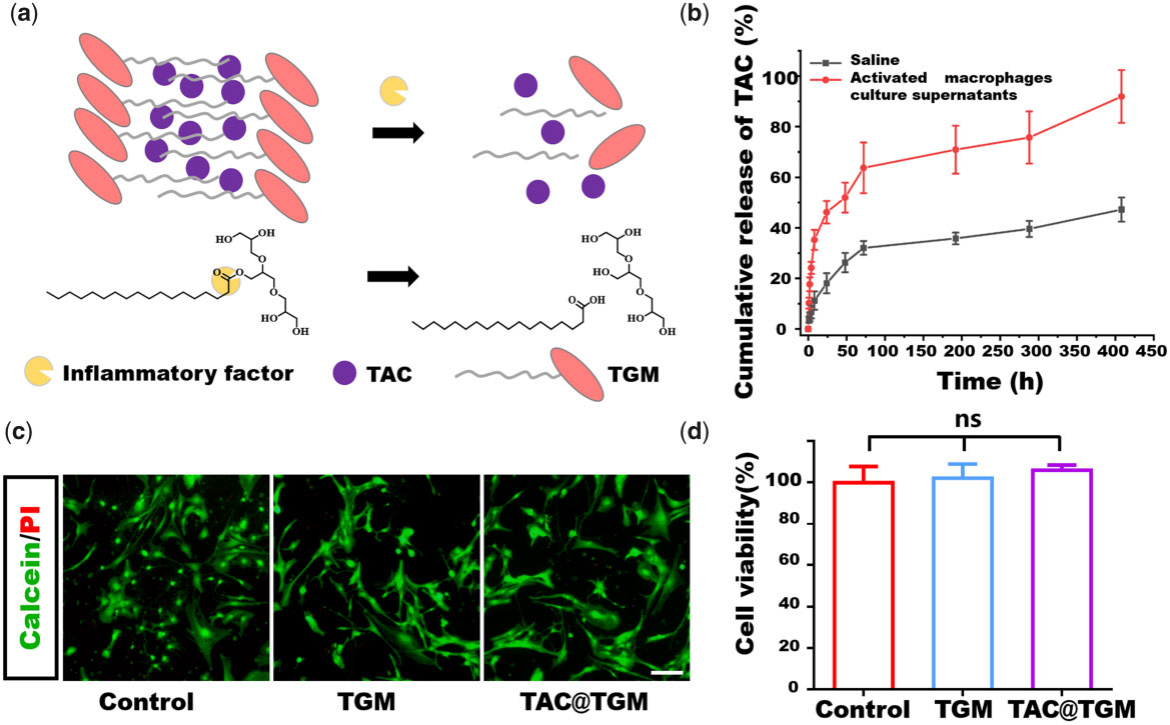
Inflammatory responsive drug release *in vitro* and cytocompatibility. (**a**) Schematic illustration of TGM hydrolyzed by inflammatory factors and releasing encapsulated TAC. (**b**)TAC release *in vitro* in the medium with or without inflammatory factors. (**c**) Live/dead staining of neural stem cells, which were incubated with culture medium or the leaching solution of TGM or TAC@TGM. Scale bar, 200 μm. (**d**) Cell viability of neural stem cells from CCK8 assay cultured with normal medium, leaching solution of TGM or TAC@TGM, ns indicated not statistically significant.

Considering that inflammatory factors can disrupt the ester bond in TGM and accelerate the release of TAC, the release kinetics of TAC was investigated *in vitro*, by incubating TAC@TGM in saline with or without activated macrophage supernatant (Fig. 2a). After 17 days, ∼47% of TAC was released in saline medium while ∼92% of TAC was released in saline with activated macrophage supernatant, indicating the high efficiency and inflammatory responsive release of TAC (Fig. 2b). The toxicity of TGM and TAC@TGM was determined by conducting live/dead cell staining and CCK8 assay *in vitro*. As shown in Fig. 2c, most of the seeded neural stem cells survived after cultured with normal medium or extracts from TGM or TAC@TGM. In the CCK8 experiments, cell viabilities in the three groups were similar, further indicating that TGM and TAC had no effect on the proliferation of nerve cells (Fig. 2d).

The daily intraperitoneal dose of TAC was determined according to the normal medication guidelines for TAC (1 mg/kg/mouse/day) [32]. Immune rejection sustained existence after SCT transplantation, high TAC drug loading in TAC@TGM could enable long-time release of TAC and the survival of transplanted tissue. And high TAC loading concentration could reduce the injection volume of TAC@TGM, which was especially pivotal for spinal tissue with narrow space around. The presented injectable TAC@TGM system achieved a TAC concentration of 50 mg/ml, which was 1 mg/ml in collagen hydrogel [33], 3 mg/ml in liposomes [34] and 10 mg/ml in supramolecular hydrogel [24] in the previous studies.

### TAC@TGM promoted functional repair of SCI after aSCT transplantation

**Figure 3.**
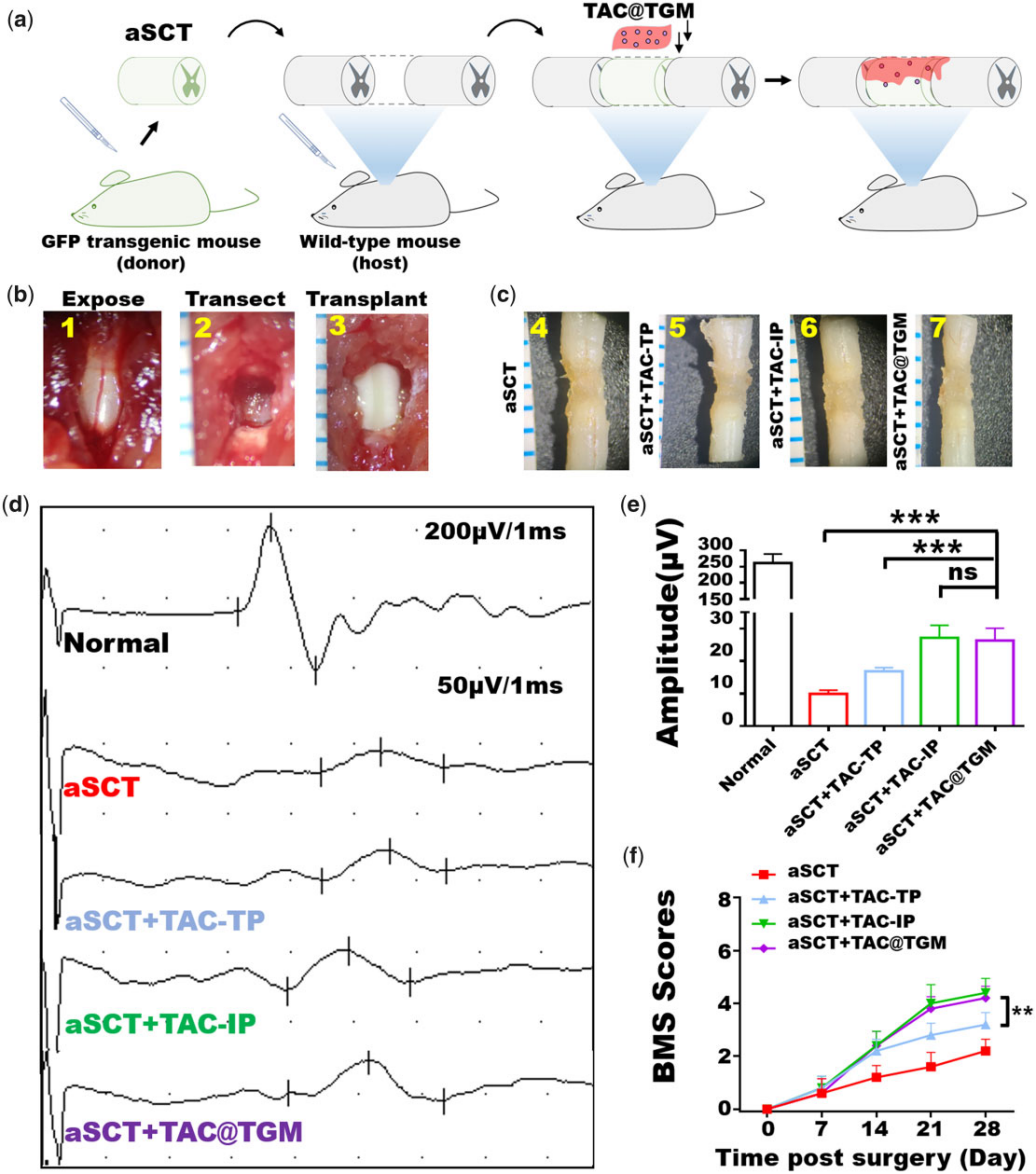
TAC@TGM facilitated electrophysiological recovery and locomotor functional improvement in aSCT transplantation mice. (**a**) Schematic diagram of aSCTs transplantation. The donated aSCTs were isolated from GFP-transgenic mice. TAC@TGM was applied to the surface of transplanted aSCT. (**b**) Surgical procedures of spinal cord transection and aSCT transplantation. 1: T7–T10 spinal cord region was exposed; 2: T8–T9 spinal cord (2 mm) was transected; 3: aSCT spinal cord was transplanted into the transection site. (**c**) Photos of spinal cord samples from each group at 4 weeks post-surgery. (**d**, **e**) MEP profiles and the amplitude ratio of MEP in the aSCT, aSCT + TAC-TP, aSCT + TAC-IP, aSCT + TAC@TGM and normal groups. (**f**) BMS scores of hind limbs from different groups during the 4-week treatment. **P < 0.01; ***P < 0.001; ns indicated not statistically significant.

Here, to visualize the survival of aSCTs, GFP transgenic mice were used as donors to track the donor tissues and cells after transplantation. Briefly, the T8–T9 spinal cords of wild-type mice were removed, and T8–T9 SCTs from adult GFP-transgenic mice were transplanted into the transection site (Fig. 3a and b). The mice (*n* = 20) were randomly divided into four groups as follows: control group with only aSCT transplantation (aSCT) (*n* = 5); aSCT + topical TAC delivery (aSCT + TAC-TP) (*n* = 5); aSCT + intraperitoneal injection of TAC (aSCT + TAC-IP) (*n* = 5) and aSCT+ TAC@TGM local delivery (aSCT + TAC@TGM) (*n* = 5). In the aSCT + TAC-IP group, TAC (1 mg/kg/day, dissolved with 5% Dimethyl sulfoxide (DMSO)/corn oil) was intraperitoneal injected daily until sacrifice; in the aSCT + TAC-TP group, 0.5 mg TAC was dissolved in 5% DMSO/corn oil and topically delivered to the surface of the transplant site; in the aSCT + TAC@TGM group, TAC@TGM containing 0.5 mg TAC was locally applied to the surface of the transplanted aSCT.

The gross morphology of spinal cords in each group showed that there was no obvious vacancy between the transplanted aSCT and the host spinal cord at 4 weeks post-surgery (Fig. 3c). And there was no significant difference among each group.

To assess the neural circuit formation in SCI mice, motor-evoked potentials (MEPs) and BMS scoring were measured at 4 weeks post-surgery (Fig. 3d–f). Mice in the aSCT + TAC-IP group (27.17 ± 3.84 μV) and the aSCT + TAC@TGM group (26.35 ± 3.78 μV) showed notable improvement of MEP in amplitudes compared with aSCT + TAC-TP group (16.85 ± 1.14 μV). Mice in the aSCT group (9.93 ± 1.15 μV) showed only slight MEP recovery. Moreover, BMS scoring was used to quantitatively evaluate whether TAC@TGM treatment could promote locomotor function recovery (Fig. 3f). Mice in the aSCT + TAC-IP and aSCT + TAC@TGM groups showed significant locomotor functional improvement, with a mean BMS score of 4.4 ± 0.55 (Supplementary Movie S1) and 4.2 ± 0.45 (Supplementary Movie S2), respectively, indicating frequent or consistent plantar stepping. Mice in the aSCT + TAC-TP group showed a lower score of 3.2 ± 0.44 (Supplementary Movie S3) than the aSCT + TAC@TGM group (*P *< 0.01), indicating plantar placing and occasional plantar stepping. Mice in the aSCT group showed only little locomotor recovery with an average score of 2.2 ± 0.45 (Supplementary Movie S4), indicating extensive ankle movement. In detail: five mice in the aSCT + TAC-IP group and four mice in the aSCT + TAC@TGM group showed more frequent crawling and more powerful lower limb contractions. Four mice in the aSCT + TAC-TP group occasionally showed strong lower limb contractions; all the transplanted mice in the aSCT group could see ankle joint movement although with weak force.

These results confirmed that aSCT transplantation could promote locomotor function recovery after SCI. However, the therapeutic outcomes were greatly limited due to the immune rejection, when compared aSCT group with other groups. One-time topical administration of TAC also could not maintain long-term effects. It is noteworthy that the locomotor function recovery in the TAC@TGM group was comparable to that in the aSCT + TAC-IP group, indicating local sustained release of TAC by TAC@TGM could achieve the similar therapeutic effects to the traditional systemic administration of TAC.

### TAC@TGM enhanced the survival of aSCT and neurons in the transplanted area

**Figure 4.**
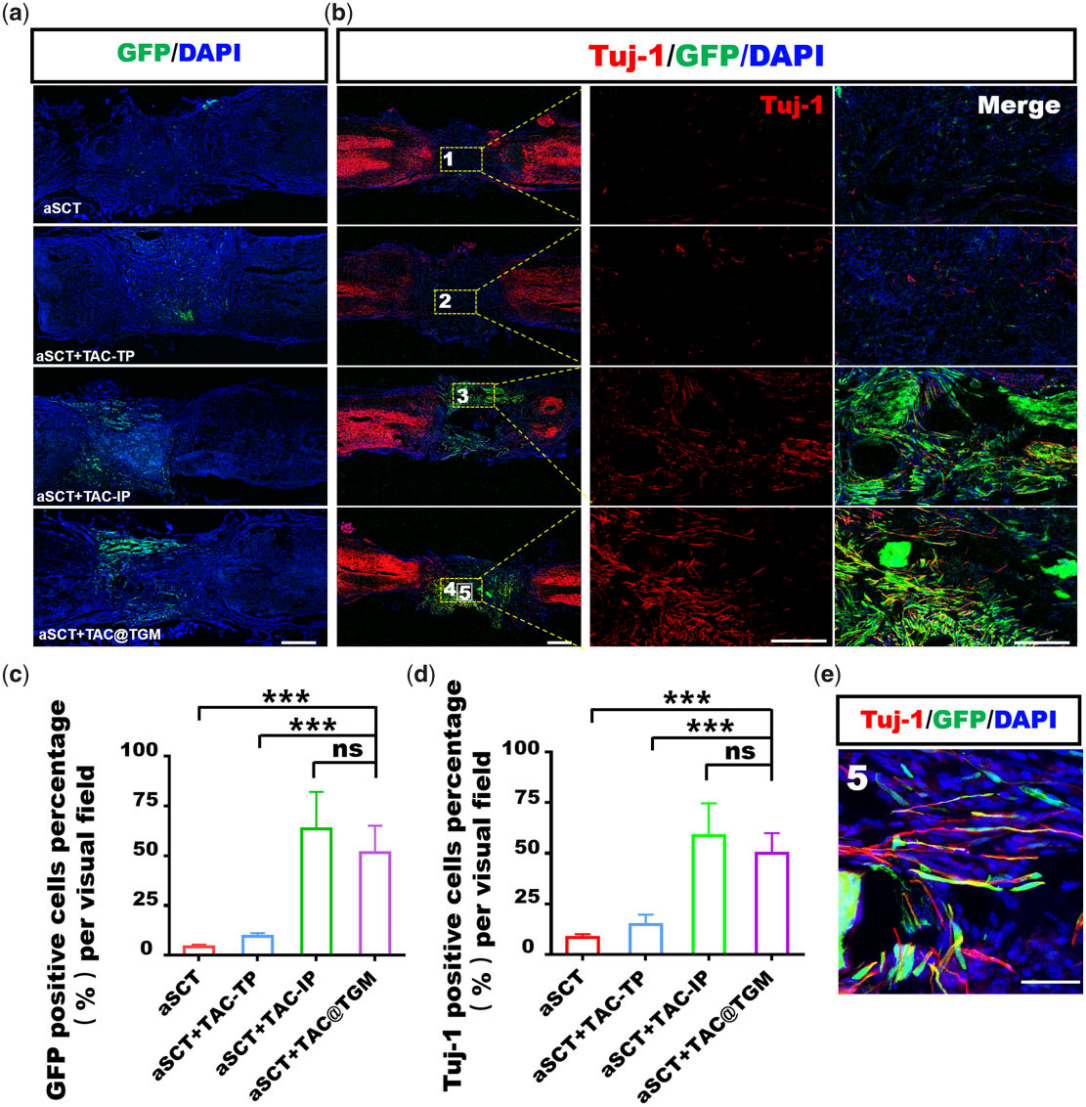
Survival of aSCT and neurons in the transplanted area at 4 weeks post-surgery. (**a**) GFP signal represented the surviving cells from donor segments in each group. Scale bar, 500 μm. (**b**) Immunofluorescent staining images of tuj-1 positive axons in each group, magnified images represent the selected area within the yellow box. Scale bars, 500 μm (left), 200 μm (middle), 200 μm (right). (**c**, **d**) Quantification of GFP positive cells and tuj-1 positive neurons in the transplant site. (**e**) Magnified images of the selected zone marked number 5 in (b) showed tuj-1 and GFP double positive neurons. Scale bar, 10 μm. ***P < 0.001, ns indicated not statistically significant.

Since TAC@TGM could promote the functional restoration of motor in aSCT-transplanted mice, the underlying mechanism was further explored. The survival of aSCT was an essential precondition to facilitate reconstruction of injured spinal cords. Neuronal survival and regeneration in the transplanted area may contribute to better locomotor function recovery [35]. Compared with aSCT and aSCT + TAC-TP groups, the survived aSCTs tissue was significantly increased at the transplanted site in the aSCT + TAC-IP and the aSCT + TAC@TGM groups as evidenced by GFP signal (Fig. 4a). To further confirm whether any interneurons survived in the transplanted site, immunofluorescence staining of the neuronal precursor marker Tuj-1 was performed. As shown in Fig. 4b, there were numerous Tuj-1 positive neurons within the transplanted spinal cord in both the aSCT + TAC-IP and the aSCT + TAC@TGM groups. More importantly, the Tuj-1 positive neurons filled the entire lesion area and almost completely connected the two transected stumps. Quantification analysis confirmed significantly enhanced percentages of GFP positive cells and Tuj-1 positive neurons in the aSCT + TAC@TGM group compared with the aSCT and aSCT + TAC-TP groups (*P *< 0.001) (Fig. 4c and d). Additionally, many Tuj-1 and GFP double positive cells existed (Fig. 4e), indicating some neurons from the donor aSCT survived after 4 weeks.

**Figure 5.**
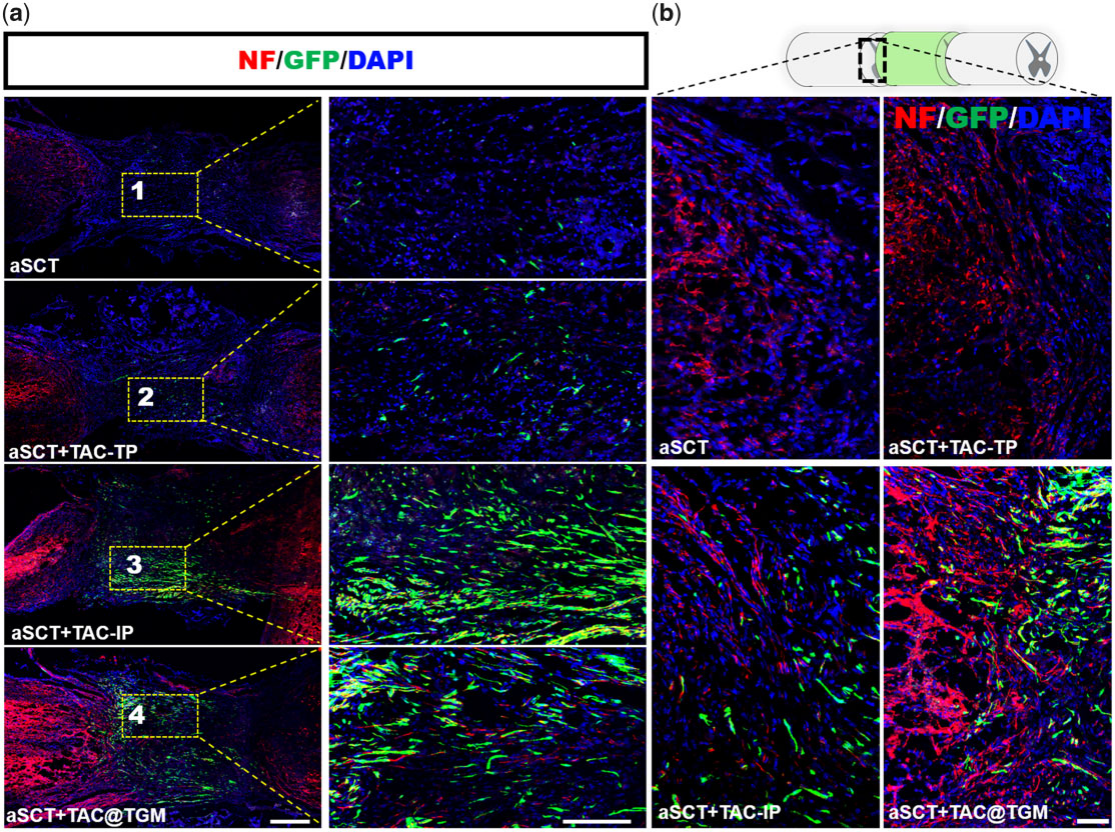
Survival NF positive neurons in the transplanted region. (**a**) NF immunofluorescence staining in the transplanted areas of each group after 4 weeks of surgery, magnified images represent the selected area within the yellow box. Scale bars, 500 μm (right), 100 μm (left). (**b**) The junction between recipient tissues and donor aSCTs in each group. Scale bars, 10 μm.

NF was another pivotal neuronal marker, here NF expression level had also been assessed in transplanted site. The results showed that a large number of NF positive neurons existed in the lesion area of mice in aSCT + TAC-IP and aSCT + TAC@TGM groups, while only a few NF positive cells were observed in aSCT +TP and aSCT groups (Fig. 5a). Quantitative analysis was consistent with the immunofluorescence observation (Supplementary Fig. S2a). As the donor–host spinal tissue integration and bridging was important for circuits reconstruction [36]. From histological view, there existed interaction between NF single positive and NF/GFP double positive neurons in aSCT + TAC@TGM group in the junction area (Fig. 5b), suggesting a good integration between donor and recipient tissues.

In summary, the locally controlled release of TAC could promote the survival of neurons in the donor aSCT, increase the total number of neurons and enhance the integration between donor and host tissue, which further promoted locomotor function recovery.

### Controlled release of TAC by TAC@TGM attenuated the local immune response

**Figure 6.**
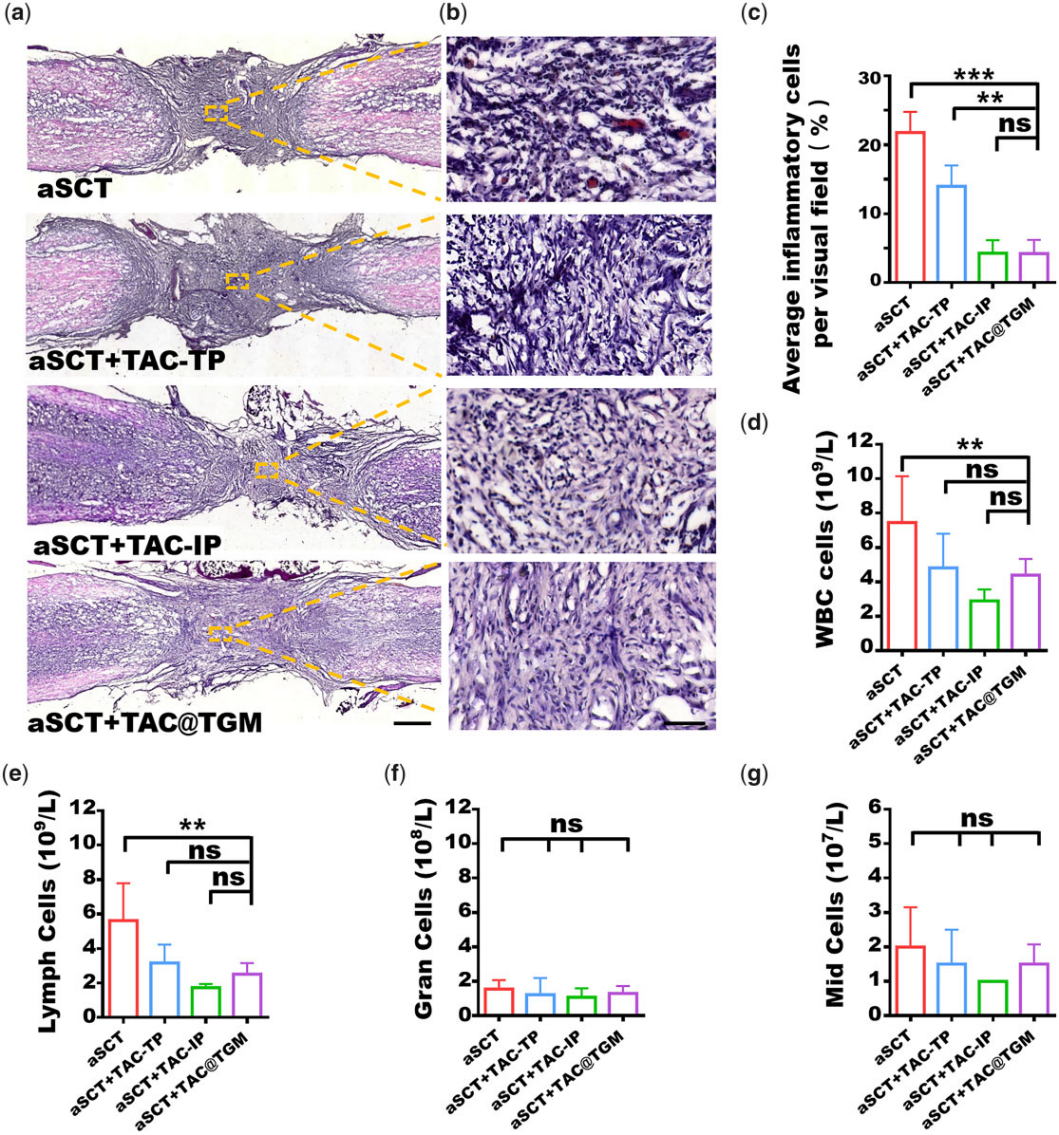
Inflammatory responses in the transplant area and blood. (**a**) HE staining of inflammatory cells in each group. Scale bars, 500 μm. (**b**) Magnified images represented the selected areas in the yellow box. Scale bars, 50 μm. (**c**) Average percentage of inflammatory cells in each group. Detection of various types of cell counts in peripheral blood including white blood cells (WBCs). (**d**) lymph cells. (**e**) Neutrophils (gran cells). (**f**) Eosinophils, basophils and monocytes (MID) cells (**g**). **P < 0.01, ***P < 0.001; ns indicates not statistically significant.

Next, the inflammatory responses were detected to explore the mechanism of TAC@TGM in promoting the survival of transplanted spinal cord. HE staining was used to analyze the infiltration of inflammatory cells into the transplant areas. Large numbers of inflammatory cells existed in the transplant area of the aSCT group and the aSCT + TAC-TP group (Fig. 6a and b). In contrast, the aSCT + TAC-IP group and the aSCT + TAC@TGM group showed significantly fewer inflammatory cells (Fig. 6c). In addition to the local inflammation, the effects of TAC@TGM on systemic blood were also measured. Blood analysis showed there were less white blood cells and lymphocytes in the aSCT + TAC-IP and aSCT + TAC@TGM groups compared with the aSCT group (Fig. 6d–g). However, the levels of leukocytes and lymphocytes in the aSCT + TAC-TP group were not statistically different from those in the aSCT + TAC@TGM group. And there was no significant difference in the numbers of eosinophils, basophils, monocytes (Mid cells) and neutrophils (Gran cells) between the four groups.

**Figure 7.**
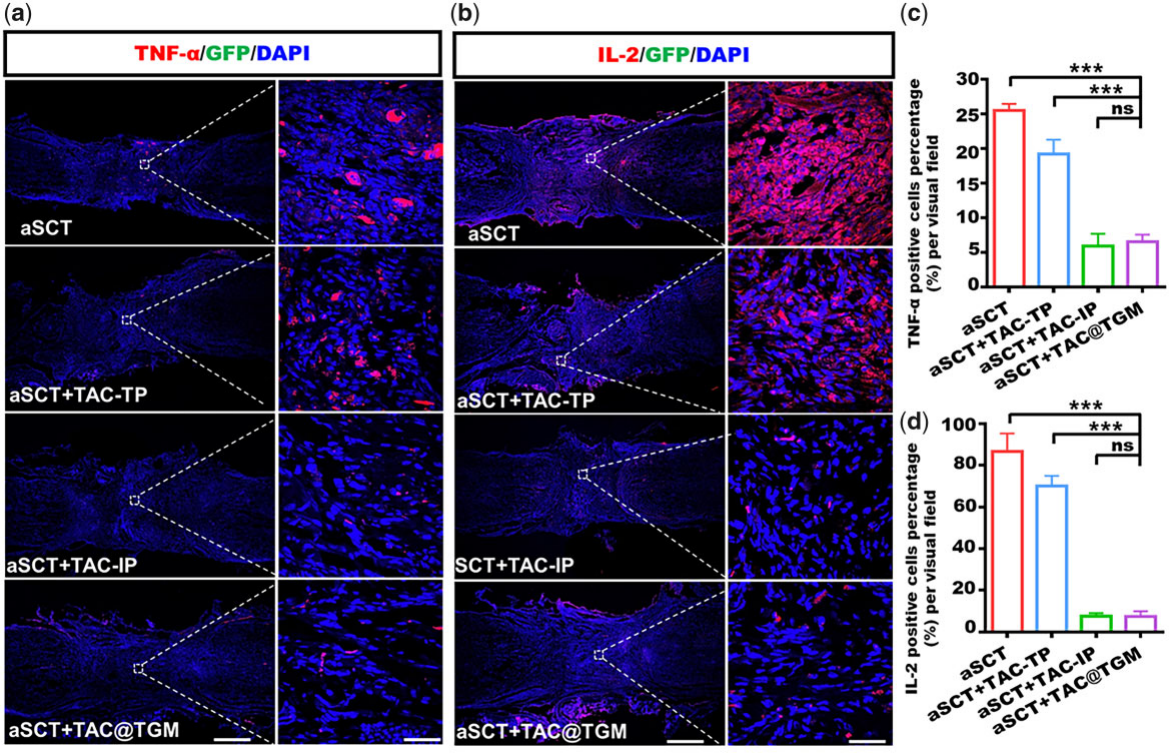
Immunofluorescence staining images of inflammatory cytokines in the transplanted area. (**a**) Immunofluorescence staining images of TNF-α in each group after 4 weeks of surgery. Scale bars, 500 μm (left) and 100 μm (right). (**b**) Immunofluorescence staining images of IL-2 T lymphocytes in each group. Scale bars represent 500 μm (left) and 25 μm (right). (**c**, **d**) Quantification of the TNF-α macrophages and IL-2 T lymphocytes in the transplanted area in each group. ***P < 0.001; ns indicated not statistically significant.

The TNF-α and cytokines IL-2 have been reported as indicators of inflammatory responses [37]. We measured the levels of TNF-α and IL-2 in the transplant area. We found there were a large amount of TNF-α and IL-2 infiltration in the graft site in the aSCT group and the aSCT + TAC-TP group (Fig. 7a and b). On the contrary, the infiltration of TNF-α and IL-2 in the aSCT + TAC-IP group and the aSCT + TAC@TGM group was very low, there was a significant statistical difference (Fig. 7c and d), indicating less local inflammatory response and anti-graft immune response.

**Figure 8.**
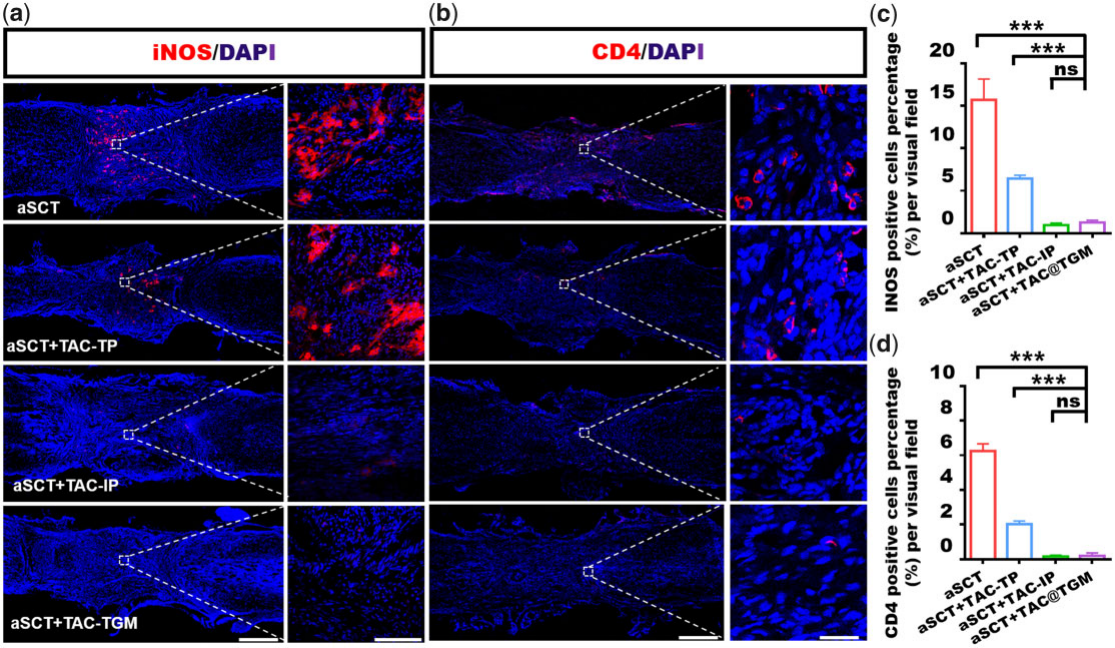
Immunofluorescence staining images of inflammatory cells in the transplanted area. (**a**) Immunofluorescence staining images of iNOS^+^ macrophages in each group after 4 weeks of surgery. Scale bars, 500 μm (left) and 100 μm (right). (**b**) Immunofluorescence staining images of CD4+ T lymphocytes in each group. Scale bars represent 500 μm (left) and 25 μm (right). (**c**, **d**) Quantification of the iNOS^+^ macrophages and CD4+ T lymphocytes in the transplanted area in each group. ***P < 0.001; ns indicated not statistically significant.

iNOS was a phenotypic marker of proinflammatory macrophages [38]. It was reported that local accumulation of proinflammatory iNOS^+^ macrophages often represented the accelerated loss of graft [39, 40]. Thus, reducing inflammatory response damage was also an important strategy to promote neural regeneration [41, 42]. The distribution of macrophages in the transplanted area was examined by immunostaining of iNOS. In this study, increased iNOS^+^ macrophages were presented in the aSCT and aSCT + TAC-TP groups compared with the aSCT + TAC-IP and the aSCT + TAC@TGM groups (Fig. 8a). CD4 was an important marker of immune rejection, representing the level of the body’s immune response [43, 44]. Immunofluorescence staining of CD4 was also performed for CD4+ T cells to evaluate the local immune rejection. In the transplanted area, decreased CD4+ T cells were found in the aSCT + TAC-IP and the aSCT + TAC@TGM group (Fig. 8b) compared with the aSCT and aSCT + TAC-TP groups. The number of iNOS^+^ cells or CD4+ T cells was not obviously different between the aSCT + TAC-IP group and the aSCT + TAC@TGM group (Fig. 8c and d).

In summary, the *in vivo* experiments confirmed that TAC@TGM could reduce local immune response after transplantation as well as reduce inflammatory response in the transplanted area, promoting the survival of the transplanted aSCT. Moreover, TAC@TGM showed a long-term protecting effect on aSCT compared with the aSCT + TAC-TP group. As for the immunosuppressive effect, TAC@TGM showed a comparable level to systemic administration, which showed great application potential for organ/tissue transplantation.

### TAC@TGM reduced the toxic effects of TAC on liver and kidneys

**Figure 9.**
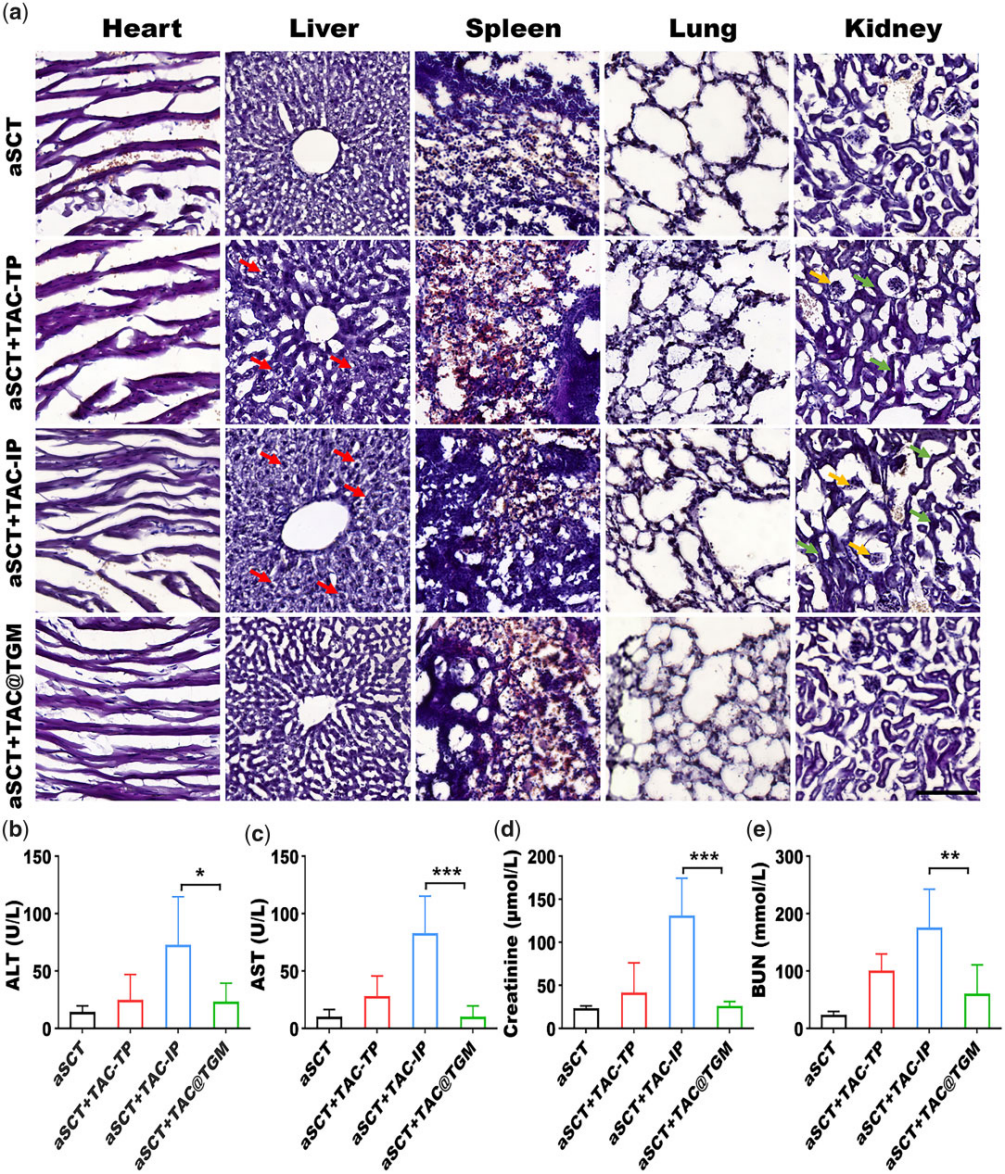
Analysis of liver and kidney injury. (**a**) HE staining images of heart, liver, spleen, lung and kidney in each above group. Red arrows indicate cytoplasmic swelling. Yellow arrows indicate atrophied glomeruli. Green arrows indicate atrophied renal tubules. Scale bars, 50 μm. Serum biochemical indicators of liver and kidney function. (**b**) ALT, (**c**) AST, (**d**) creatinine, (**e**) BUN. *P < 0.05, **P < 0.01, ***P < 0.001; ns indicated not statistically significant.

TAC has been reported to have toxic effects on various organs [12, 45]. It induced renal and liver dysfunction by inducing apoptosis [46], and generating reactive oxygen species [47], which caused oxidative stress and mitochondrial dysfunction [48]. Here, HE staining was performed to find out whether local release of TAC could reduce the toxic effects on other organs. The heart, spleen and lung showed normal histological structures in the four groups with no significant differences. Liver is the main metabolic and detoxification organ for most drugs [49], the effect of TAC on the liver was obvious. Massive hepatocytes with cytoplasmic swelling were observed in the aSCT + TAC-IP and aSCT + TAC-TP groups. In addition, kidney as a metabolic organ was also affected [15], glomerular and tubular atrophy were observed in the kidneys in the aSCT + TAC-IP and aSCT + TAC-TP groups (Fig. 9a). While liver and kidney of the aSCT + TAC@TGM group exhibited normal histological appearance. These results were consistent with earlier studies, which reported that obvious toxic effects of high-dose (aSCT + TAC-TP group) or long-term systemic use (aSCT + TAC-IP group) of TAC on liver and kidney [18, 50].

ALT and AST are sensitive indicators of liver injury, which tend to increase when liver injury occurs, indicating that liver function is impaired [51]. Creatinine and BUN were the metabolites of the body, which were excreted through the kidneys. The serum levels of creatinine and BUN increased when renal function was abnormal [52]. We had detected ALT, AST, creatinine and BUN as quantitative biochemical indicators to monitor hepatic and renal toxicity of TAC in mice. The results showed that aSCT + TAC@TGM group had significantly lower serum levels of ALT (*P* < 0.05), AST (*P* < 0.001), creatinine (*P* < 0.001) and BUN (*P* < 0.01) than aSCT + TAC-IP group (Fig. 9b–e), suggesting that the liver and kidney injury caused by TAC was more serious in aSCT + TAC-IP group. TAC@TGM reduced the toxic effect of TAC on liver and kidney injury through local TAC delivery. These results indicated that this local TAC delivery system had more advantage in reducing complications caused by systemic administration of TAC.

## Conclusion

Here, we successfully developed a TGM-based local controlled release system for TAC (TAC@TGM) by using a facile two-step method. The *in vitro* experiments demonstrated that TAC@TGM was injectable with high drug loading efficiency, and inflammatory-responsive release of TAC. The *in vivo* experiments demonstrated that TAC@TGM could effectively reduce the infiltration and activation of inflammatory cells and promote the survival and function of transplanted aSCT. The TAC@TGM could reach a comparable therapeutic effect to traditional administration of TAC, while alleviate the toxic effects of TAC on liver and kidneys. In summary, this study presented an effective encapsulation and release system of immunosuppressant TAC, which was also potential for the delivery of hydrophobic drugs. In addition, this localized immunosuppressive system provided a potential strategy to suppress immunorejection of tissue/organ transplantation as well as cell transplantation in clinic.

## Supplementary Material

rbac097_Supplementary_Data
