# Supplementary material for: Locally controlled release of immunosuppressive promotes survival of transplanted adult spinal cord tissue
Source: Regen Biomater. 2022 Dec 5;10:rbac097. doi: 10.1093/rb/rbac097 (PMC9845520; doi:10.1093/rb/rbac097)
Supplement: rbac097_Supplementary_Data [file rbac097_supplementary_data.zip › rbac097_Supplementary_Data/Supplementary Material-final updates.docx]

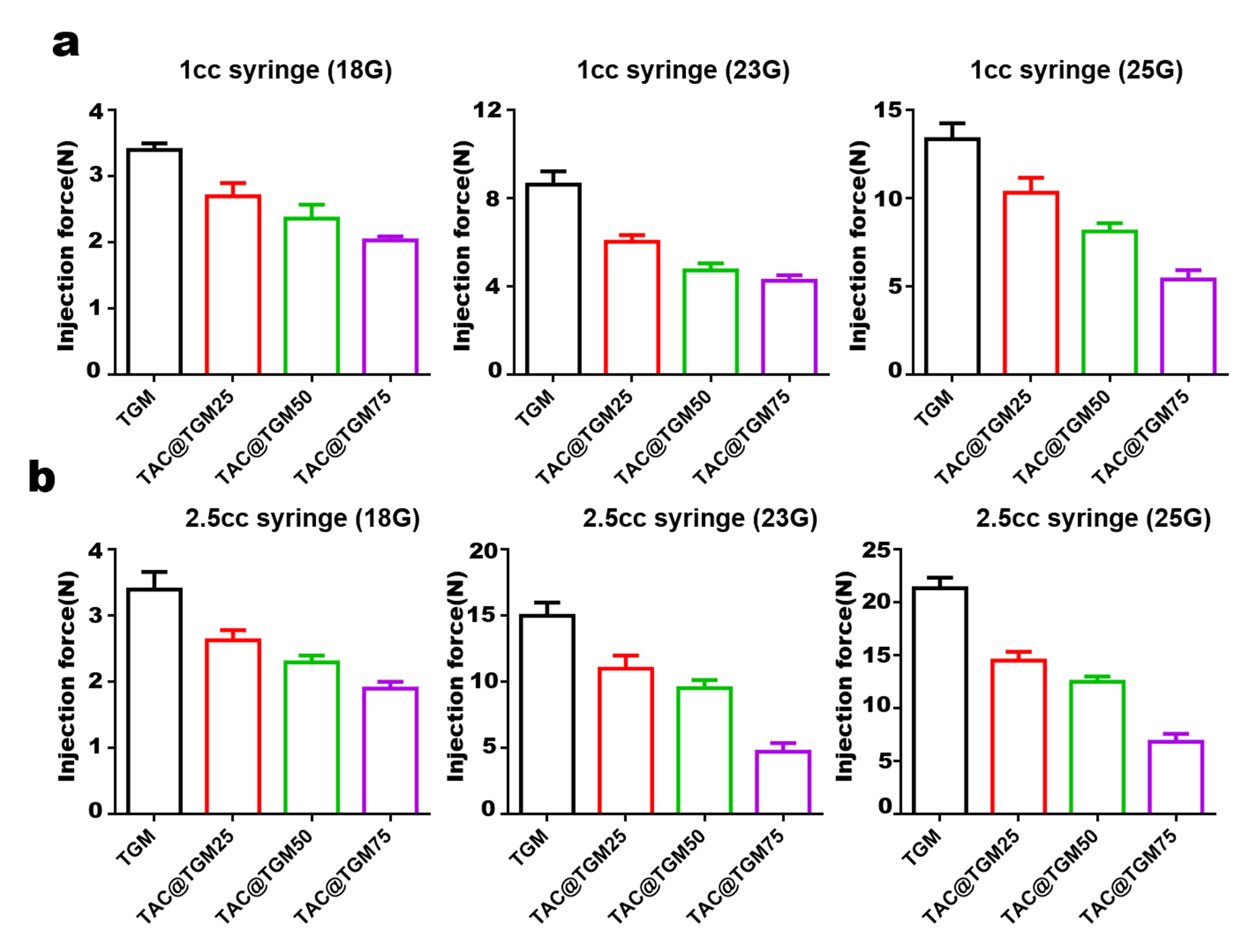


**Supplementary Figure 1.** Injectability of TAC@TGM through various syringe and needles. (a) a 1cc syringe or (b) 2.5cc syringe with different sizes of catheters.


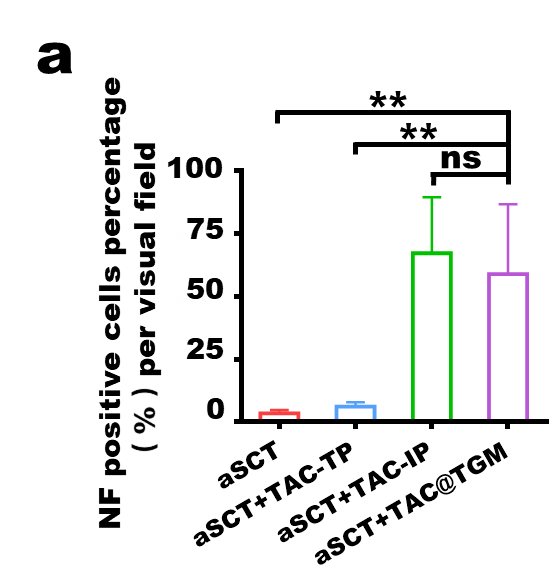


**Supplementary Figure 2.** Survival NF positive neurons in the transplanted region. (a) Quantification of NF positive neurons in the transplant area. ** *p* < 0.01, ns indicated not statistically significant.
